# Supplementary material for: Comparative analysis and FPGA realization of different control synchronization approaches for chaos-based secured communication systems
Source: PLoS One. 2024 Jan 23;19(1):e0291714. doi: 10.1371/journal.pone.0291714 (PMC10824461; doi:10.1371/journal.pone.0291714)
Supplement: Attachment — Submitted filename: Response to Reviewers.docx [file pone.0291714.s003.docx]

**Journal	:** PlOS ONE

**Paper Title	:** Comparative Analysis and FPGA Realization of Different Control Synchronization Approaches for Chaos-based Secured Communication Systems

**Paper ID	:** PONE-D-22-28879

**Authors:**

We acknowledge the Review Reports received on our submission to Plos One Journal. We have carefully revised our manuscript according to the suggestions given by Reviewers 1, 2, 3 and 4. Our specific responses to review comments on Plos One Journal are detailed below.

Reviewer 1:

The refs of the paper is inadequate, it is suggested that the author cite some relevant refs, i.e. 1. Ye X., Wang X., Gao S., et al.: A new chaotic circuit with multiple memristors and its application in image encryption. Nonlinear Dynamics, 99, 1489-1506 (2020). 2. Ye, X.L., Mou, J., Luo, C.F., Wang, Z.S.: Dynamics analysis of Wien-bridge hyperchaotic memristive circuit system. Nonlinear Dynamics, 92, 923-933 (2018). 3. B. Liu, X. Ye, and G. Hu, “Design of a New 3D Chaotic System Producing Infinitely Many Coexisting Attractors and Its Application to Weak Signal Detection,” INTERNATIONAL JOURNAL OF BIFURCATION AND CHAOS, vol. 31, p. 2150235, 2021. 4. S. Xu, X. Wang , X. Ye, “A new fractional-order chaos system of Hopfield neural network and its application in image encryption,”Chaos, Solitons & Fractals, Volume 157, April 2022, 111889.

**Authors:**

Many thanks for your comment. We have checked carefully refs suggestion, it is relevant work with our paper. We have added these refs in new version in introduction, kindly check red highlight.

[1a] Ye, X., Wang, X., Gao, S., Mou, J., Wang, Z., & Yang, F. (2020). A new chaotic circuit with multiple memristors and its application in image encryption. Nonlinear Dynamics, 99(2), 1489-1506.

[1b] Ye, X., Mou, J., Luo, C., & Wang, Z. (2018). Dynamics analysis of Wien-bridge hyperchaotic memristive circuit system. Nonlinear Dynamics, 92(3), 923-933.

[1c] Liu, B., Ye, X., & Hu, G. (2021). Design of a New 3D Chaotic System Producing Infinitely Many Coexisting Attractors and Its Application to Weak Signal Detection. International Journal of Bifurcation and Chaos, 31(15), 2150235.

[1d] Xu, S., Wang, X., & Ye, X. (2022). A new fractional-order chaos system of Hopfield neural network and its application in image encryption. Chaos, Solitons & Fractals, 157, 111889.

**Reviewer 1:**

In this paper, the format is not uniform of each paragraph. The author should check every section carefully.

**Answer:**

Many thanks for your comment. We have fix them in new version.

**Reviewer 1:**

The paper should be reorganized. In the current form, it seems like some related sections. For example, sections 1 and 2 should be combined, sections 6 and 7 should be combined.

**Answer:**

Thanks for your good comment. Reviewer is correct, we have reorganized of this paper with good standar in new version.

**Reviewer 1:**

The coordinates of all figures are too small. In Figure 3, the layout of the picture is not beautiful.

Answer

Many thanks for your comment. Reviewer is right. We have displayed with large Figure in new version.

**Reviewer 1:**

In this paper, u(i) is a control signal. In Figure 5-13, it is not standard to use u(i) to replace x(i) and y(i). The meaning of u(i) is also not explained in Figure.

Answer

We thank the reviewer for pointing out this important issue. In the mentioned Figures we used Matlab Function which deals with the input signals based on their number u(1), u(2), … u(i) instead of signals names. To address this comment, we added the following descriptions:

Figure 5 illustrates the Matlab/Simulink representation of the master blocks of the 4-D hyperchaotic system given in the equations (1.1). The master system consists of four main parts, one for each state variable. Each part has four inputs u(1), u(2), u(3), and u(4) represents state variable vector of the master system[x1, x2, x3, x4]. Similarly, Figure 6 shows the Matlab/Simulink representation of the slave blocks of the 4-D hyperchaotic system given in the equations (3.1). In this Figure u(1), u(2), u(3), and u(4) represents state variable vector of the slave system [y1, y2, y3, y4], while u(5), u(6), u(7), and u(8) represents the control vector [u1, u2, u3, u4] that keeps the slave system on the desired trajectory.

We also added the following details to the Figures 7 , 8, and 10:

The Matlab/Simulink representation of the parameter estimation block shown in Figure 7 indicates the integration of parameter update laws in (3.9) based on the error between the slave and the master systems. Where [u(1), u(2), u(3), u(4)] and, [u(5), u(6), u(7), u(8)] represent state variable vectors of the master and slave system respectively.

The representation of the Adaptive controller block is shown in Fig 8. The block indicates the derived Adaptive control laws based on (3.3) and the values of parameters from the estimated parameters block. Where [u(1), u(2), u(3), u(4)] represent the error vector [e_1, e_2, e_3, e_4], [u(5), u(6), u(7), u(8)], [u(9), u(10), u(11), u(12)] represent state variable vectors of the master and slave system respectively, and [u(13), u(14)] represent the estimated parameters [A1 ,A2].

The representation of the LQR block is shown in Figure 11. The block indicates the 8.derived control law based on (3.11) and the gain matrix K of the LQR controller. Where u(1) and u(3) represent x(1) and x(3) state variables of the master system. While u(5) and u(7) represent y(1) and y(3) state variables of the slave system.

Finally, the following description is added to Figure 11

Figure 13 shows the top-level block diagram for the synchronization of 4-D system. The block diagram consists of three basic blocks, the master system where the output of this system is [x1, x2, x3, x4], the controller which compares between the master and slave system to generate the control vector [u1, u2, u3, u4], and the slave system which receive the control vector to synchronous the master system. where the output vector of the slave system is [y1, y2, y3, y4].

**Reviewer 1:**

What is the difference between Figure 9, Figure 12 and Figure 19? In my opinion, Figure 19 can replace Figure 9 and Figure 12

Answer

We thank the reviewer for this comment. To address this comment we have deleted Figures 9, 12. We also added the following details to Figure 19 (Now it becomes Figure 17 after deleting Figures 9 and 12)

Figures 17a, 17b, 17c and 17d illustrate the state space synchronization using two control methods, while Figure 17e shows a comparison of the synchronization error for the two methods. Where the reference signals are marked in blue color, the responses using LQR and adaptive control are marked with green and red colors respectively. Each signal starts from its initial value till the matching between the master and slave trajectories is achieved by the LQR.

**Reviewer 1:**

In this paper, an implementation of two different control approaches for synchronization between two identical four-dimensional hyperchaotic systems is presented. Why should author compare the synchronization performance of the two methods through the three-dimensional system

Answer

We would like to thank the respected reviewer for this comment. We apply the synchronization methods on the 3-D chaotic system to make sure that we will obtain the same results for different types of chaotic systems. To address this comment, we add the following paragraph to Section 6.

In this section, we will apply both synchronization techniques to the 3-D chaotic system in order to draw conclusions about the results of the comparison while also ensuring that the outcomes are consistent across various kinds of chaotic systems.

**Reviewer 1:**

For the adaptive control approach, author gives a definite controller about four-dimensional hyperchaotic system. However, LQR only puts forward a design scheme. This paper should give definite controller, the true matrix form of Q, R and K. And, above all, for the adaptive control approach, the controller proposed in this paper for four-dimensional systems is not applicable to three-dimensional systems.

Answer

Thank you for this comment. To address this comment, we have added the following paragraph to the Section 3.2:

For the 4-D hyperchaotic system, we choose Q=100.I4×4 and R=I4×4. Now, the control law could be achieved by solving the Ricatti equation in MATLAB using the 'lqr' command to determine P and K matrices.

$$P=K=\left[ \begin{matrix} 9.0442 & 0.4494 & \begin{matrix} 0.0000 & 0.0449 \end{matrix} \\ 0.4494 & 10.0348 & \begin{matrix} 0.0000 & 0.0212 \end{matrix} \\ \begin{matrix} 0.0000 \\ 0.0449 \end{matrix} & \begin{matrix} 0.0000 \\ 0.0212 \end{matrix} & \begin{matrix} \begin{matrix} 10.0000 \\ 0.0000 \end{matrix} & \begin{matrix} 0.0000 \\ 9.9954 \end{matrix} \end{matrix} \end{matrix} \right]$$

We also added the following paragraph to Section 6:

For the adaptive controller of the 3-D chaotic system, we take the gain constants as ki=5 for i=1,2,3. For the LQR, we choose Q=100.I4×4 and R=I4×4. Thus, the control law could be achieved by solving the Ricatti equation in MATLAB using the 'lqr' command to determine P and K matrices.

$$P=K=\left[ \begin{matrix} 9.7626 & 9.1315 & 0.0000 \\ 9.1315 & 14.1154 & 0.0000 \\ 0.0000 & 0.0000 & 9.0499 \end{matrix} \right]$$

**Reviewer 2**

Some parameters have not been defined in the paper. The authors should define the missed parameters.

**Authors:**

We thank the reviewer for the comment. We have define variable equation in the new version

**Reviewer 2:**

Background study about secure communication is very low in introduction

**Answer:**

We thank the reviewer for the comment. We have added the literature for the secure communication system in the new version.

[1e] Sambas, A., Vaidyanathan, S., Zhang, X., Koyuncu, I., Bonny, T., Tuna, M., ... & Kumam, P. (2022). A novel 3D chaotic system with line equilibrium: multistability, integral sliding mode control, electronic circuit, FPGA implementation and its image encryption. IEEE Access, 10, 68057-68074.

**Reviewer 2:**

What is the control objective for this work?

Answer

The main objective of this control is design and derivation of two control approaches (Adaptive and LQR) for synchronization of the proposed hyperchaotic. Furthermore, the compare the performance of two controllers in the synchronization of the proposed hyperchaotic system. Finally, generalizing comparison result by applying the synchronization on another family of chaotic systems using Adaptive control and LQR.

**Reviewer 2:**

Please check again all equation

We thank the reviewer for the comment. We have checked and correction for all equation in new version

**Reviewer 2;**

What are the advantages of the proposed control system?

Answer

We thank the reviewer for the comment. For this problem, we have explained about the advantage of control system in Section 6 and Section 7.

Reviewer 2

The main problem considered in this paper should be further described.

Answer

We gratefully appreciate for your valuable suggestion. One of the important challenges in designing a chaos-based secured communication system is the synchronization between the transmitter and the receiver oscillations when the initial conditions change. Because chaotic systems show high sensitivity to initial conditions, so the synchronization of chaotic systems seems to be application in secure communication system.

Reviewer 2

Future works should be added to the last of the conclusions.

Answer

We gratefully appreciate for your valuable suggestion. We have added future research in conclusion.

Reviewer 2

Check typo error and grammatical error

Answer

We gratefully appreciate for your comment, we have correction typo and grammatical error in new version.
