# Supplementary material for: Comparative analysis and FPGA realization of different control synchronization approaches for chaos-based secured communication systems
Source: PLoS One. 2024 Jan 23;19(1):e0291714. doi: 10.1371/journal.pone.0291714 (PMC10824461; doi:10.1371/journal.pone.0291714)
Supplement: Attachment — Submitted filename: my comments.docx [file pone.0291714.s004.docx]

Comments on: PONE-D-23-16430_reviewer.

This paper clearly demonstrates the incorrect signal recovery caused by variations of initial conditions and state parameters, proposes two different control approaches with stability analysis and performance comparison stating the pros and cons. I would suggest publication of this paper after solving my following questions:

(1) Please fix typos and format incompatibility issues, such as question mark at citation number 8 and equation 4.10 (derivative of e instead of e).
(2) I also published one analog implementation (<https://dergipark.org.tr/en/download/article-file/2772051>) of a nonlinear system demonstrating chaos via FPAA, is there any reasons why you choose digital system implementation instead of analog system implementation?
(3) Your variable u is used to represent both control inputs and system states, which is very confusing, I would recommend changing them to two different representations.
(4) Could you show some simulation results comparing the performance between adaptive controller and LQR controller? As I could not find it anywhere in your papers.
(5) Your controller is initially designed to work with a 4-D system, and then you used a 3-D system to demonstrate the robustness of your proposed controllers, while, how can you directly apply the same controller to two different systems with different dimensions? Could you explain more details on this?
