# Supplementary material for: Comparative analysis and FPGA realization of different control synchronization approaches for chaos-based secured communication systems
Source: PLoS One. 2024 Jan 23;19(1):e0291714. doi: 10.1371/journal.pone.0291714 (PMC10824461; doi:10.1371/journal.pone.0291714)
Supplement: Attachment — Submitted filename: Responces.docx [file pone.0291714.s005.docx]

**Journal	:** PlOS ONE

**Paper Title	:** Comparative Analysis and FPGA Realization of Different Control Synchronization Approaches for Chaos-based Secured Communication Systems

**Paper ID	:** PONE-D-22-28879

**Authors:**

We acknowledge the Review Reports received on our submission to Plos One Journal. We have carefully revised our manuscript according to the suggestions given by the Reviewer. Our specific responses to review comments on Plos One Journal are detailed below.

**This paper clearly demonstrates the incorrect signal recovery caused by variations of initial conditions and state parameters, proposes two different control approaches with stability analysis and performance comparison stating the pros and cons. I would suggest publication of this paper after solving my following questions:**

**Ans.** The authors would like to thank the respected reviewer for your valuable feedback and attention to detail in reviewing our manuscript.

(1) **Please fix typos and format incompatibility issues, such as question mark at citation number 8 and equation 4.10 (derivative of e instead of e).**

**Ans.** We appreciate your effort in identifying the typographical errors and format incompatibility issues in our work. This typos error has been corrected.

(2) **I also published one analog implementation (** [**https://dergipark.org.tr/en/download/article-file/2772051**](https://dergipark.org.tr/en/download/article-file/2772051) **) of a nonlinear system demonstrating chaos via FPAA, is there any reasons why you choose digital system implementation instead of analog system implementation?**

**Ans.** We highly regard analog implementations' insights into nonlinear dynamics, yet our choice of a digital path aligns with our study's emphasis on meticulous analysis, systematic exploration, and comparative assessment within a controlled and measurable framework. To address this concern, we add the following to Section 1:

In this paper we decided to use digital implementation using FPGA, even though we appreciate the value in using analog implementation using Field-Programmable Analog Array FPAA [42]. Digital tools allow us to repeat experiments more accurately, which is important when we want to see how our control method interacts with nonlinear systems. Digital systems let us compare different ways of controlling things directly, so we can see how well our approach works compared to other methods. The clarity of documentation and data recording in digital implementations aids transparent and replicable reporting of our methodology and results.

[42] Li Xiaofu, BEAL Aubrey, DEAN Robert, and Edmon Perkins. Chaos in a pendu-lum adaptive frequency oscillator circuit experiment. Chaos Theory and Applications,5(1):11–19, 2023.

(3) **Your variable u is used to represent both control inputs and system states, which is very confusing, I would recommend changing them to two different representations.**

**Ans.** We acknowledge your observation regarding the confusion caused using the variable "u" to represent both control inputs and system states. To address this concern, we make the following figures has been updated; Figure 5, Figure 6, Figure 7, Figure 8, and Figure 10.

Figure 5 illustrates Matlab/Simulink representation of the master blocks of the 4-D hyperchaotic system given in equations (2.1). The master system consists of four main parts, one for each state variable where x(1), x(2), x(3), and x(4) represents the state variable vector of the master system. Similarly, Figure 6 shows the Matlab/Simulink representation of the slave blocks of the 4-D hyperchaotic system given in equations (4.1).

In this Figure y(1),y(2),y(3),y(4) represents the state variable vector of the slave system, while u(1),u(2),u(3),u(4) represents the control vector that keeps the slave system on the desired trajectory.

The Matlab/Simulink representation of the parameter estimation block shown in Figure 7 indicates the integration of parameter update laws in (4.9) based on the error between the slave and the master systems. Where in Figure 7 x(1), x(2), x(3) ,x(4) and, y(1), y(2), y(3), y(4) represent state variable vectors of the master and slave system respectively. The representation of the Adaptive controller block is shown in Figure 8. The block indicates the derived Adaptive control laws based on (4.3) and the values of parameters from the estimated parameters block.

The representation of the LQR block is shown in Figure 10. The block indicates the derived control law based on (4.11) and the gain matrix K of the LQR controller.

(4) **Could you show some simulation results comparing the performance between adaptive controller and LQR controller? As I could not find it anywhere in your papers.**

**Ans.** The authors would like to thank the respected reviewer for rising this comment.

Actually Figures 17a-17e in Section 6, indeed provides valuable insights into the simulation results for the state space synchronization and presents a glimpse of the performance of the two methods. Moreover, this section includes detailed comparison between the two synchronization methods based on different indicators.

We also welcome any additional insights or details you, as the reviewer, might possess regarding the comparative process. Your expertise in this area could greatly enhance the depth of our analysis and further refine the accuracy of our presentation. We are committed to ensuring a thorough and accurate comparison that addresses your valuable concerns.

(5) **Your controller is initially designed to work with a 4-D system, and then you used a 3-D system to demonstrate the robustness of your proposed controllers, while, how can you directly apply the same controller to two different systems with different dimensions? Could you explain more details on this?**

**Ans.** The authors would like to thank the respected reviewer for rising this important comment. We understand the importance of providing clarity on this aspect. To address this, we have added the following paragraph to Section 2

The process of applying the same controller to systems with varying dimensions involves careful consideration of the underlying dynamics and control architecture. In our case, while the initial design was tailored for a 4-D system, the fundamental control principles remain applicable to systems with lower or higher dimensions.
